# On Bougies of Slippery Elm Bark

**Published:** 1845-09

**Authors:** Wm. B. Herrick

**Affiliations:** Prof. of Anatomy in the Rush Medical College


*On Bougies of Slippery Elm Bark.* By Wm. B. Herrick, M. D., Prof. of Anatomy in the Rush Medical College.

Strictures of the urethra, rectum, œsophagus, &c., are often, to the surgeon, most obstinate and troublesome diseases, calling into requisition experience, skill, and the use of every known means for their palliation and relief.

Numerous instruments, for dilating, dividing and cauterising the walls of such contracted passages, have been invented, and are in the hands of some of our most experienced surgeons; yet cases of this kind are frequently met with, by country practitioners, who, for the want of such instruments, fail, or find themselves greatly perplexed in their treatment.

It is also true, that surgeons well provided with metallic and gum elastic bougies and catheters, meet with many cases in which the irritation produced by them precludes the possibility of allowing these irritating and unyielding substances to remain sufficiently long in the canal to dilate permanently the contracted portion; and in some cases an instrument of sufficient diameter cannot be introduced.

In view of the above facts, it is considered not inexpedient to call the attention of physicians generally to the merits of the *slippery-elm bougie;* a simple, but, in many cases, very efficient instrument, not in general use, but one, the utility of which is not to be doubted.

This bougie should be made of the firm inner bark of the elm, of a form similar to that of a gum elastic bougie or catheter, and of a size adapted to the case under treatment. The facility with which any practitioner can, from this very common material, supply himself with such an instrument, is evidently one of its great advantages. It is superior to others, also, from the fact that the moistened mucilaginous surface of this bark is emollient, and soothing, and tends to diminish rather than to increase irritation. Its greatest merit, however, is that of being expanded by the natural moisture of the part, when introduced, in the dry state, into a secreting passage. Its peculiarity in this respect enables the surgeon to overcome strictures without force, by means of the gradually applied expansive properties of the instrument, which can be made, therefore, sufficiently small to admit of easy introduction. By using these bougies, one after another, gradually increasing their size, the required diameter can be given, by the operator, to most contracted passages. From the fact also, that that part of the instrument remaining in the constricted portion of the canal during its dilatation by moisture, is less expanded than the uncompressed portions, the surgeon can, upon withdrawing it, judge, by its shape, of the degree of firmness in the part, and determine at once the exact situation and extent of a stricture.

The utility of this simple instrument, as tested by the writer in strictures of the urethra; for dilating that canal after cutting for stone, and as a tent to sooth and prevent the closure of passages leading to suppurating cavities; are such as fully to justify him, in thus calling the attention of the profession to its merits.

